# Update on poly(ADP-ribose) polymerase inhibitors resistance in ovarian cancer

**DOI:** 10.3389/fphar.2023.1164395

**Published:** 2023-06-23

**Authors:** Ruihong Dong, Ting Ding, Zhengyu Li

**Affiliations:** ^1^ Department of Gynecology and Obstetrics, West China Second University Hospital, Sichuan University, Chengdu, China; ^2^ Key Laboratory of Birth Defects and Related Diseases of Women and Children (Sichuan University), Ministry of Education, Chengdu, China

**Keywords:** poly ADP-ribose polymerase inhibitors, ovarian cancer, drug resistance, mechanism, combination therapy

## Abstract

Ovarian cancer is one of the most common reproductive system tumors. The incidence of ovarian cancer in China is on the rise. Poly(ADP-ribose) polymerase (PARP) inhibitor (PARPi) is a DNA repair enzyme associated with DNA damage repair. PARPi takes PARP as a target to kill tumor cells, especially for tumors with homologous recombination (HR) dysfunction. Currently, PARPi has been widely used in clinical practice, mainly for the maintenance of advanced ovarian epithelial cancer. The intrinsic or acquired drug resistance of PARPi has gradually become an important clinical problem with the wide application of PARPi. This review summarizes the mechanisms of PARPi resistance and the current progress on PARPi-based combination strategies.

## 1 Introduction

Ovarian cancer (OC) is one of the most common gynecological malignancies. OC has the worst prognosis and the highest mortality. Over 19,000 new cases of OC and 12,000 deaths were estimated in 2022 in the United States alone according to the American Cancer Society (Siegel et al., 2022). OC is the seventh most common type of malignant tumor in women and the eighth cause of mortality in them worldwide (Gaona-Luviano et al., 2020). Early-stage patients have a better prognosis, but most patients are diagnosed at an advanced stage. Epithelial OC accounts for about 80% in advanced-stage patients. Surgical debulking and platinum-based chemotherapy (such as carboplatin and paclitaxel) are recognized first-line treatment regimens. Yet, the long-term results of these treatments are not satisfactory.

**FIGURE 1:**
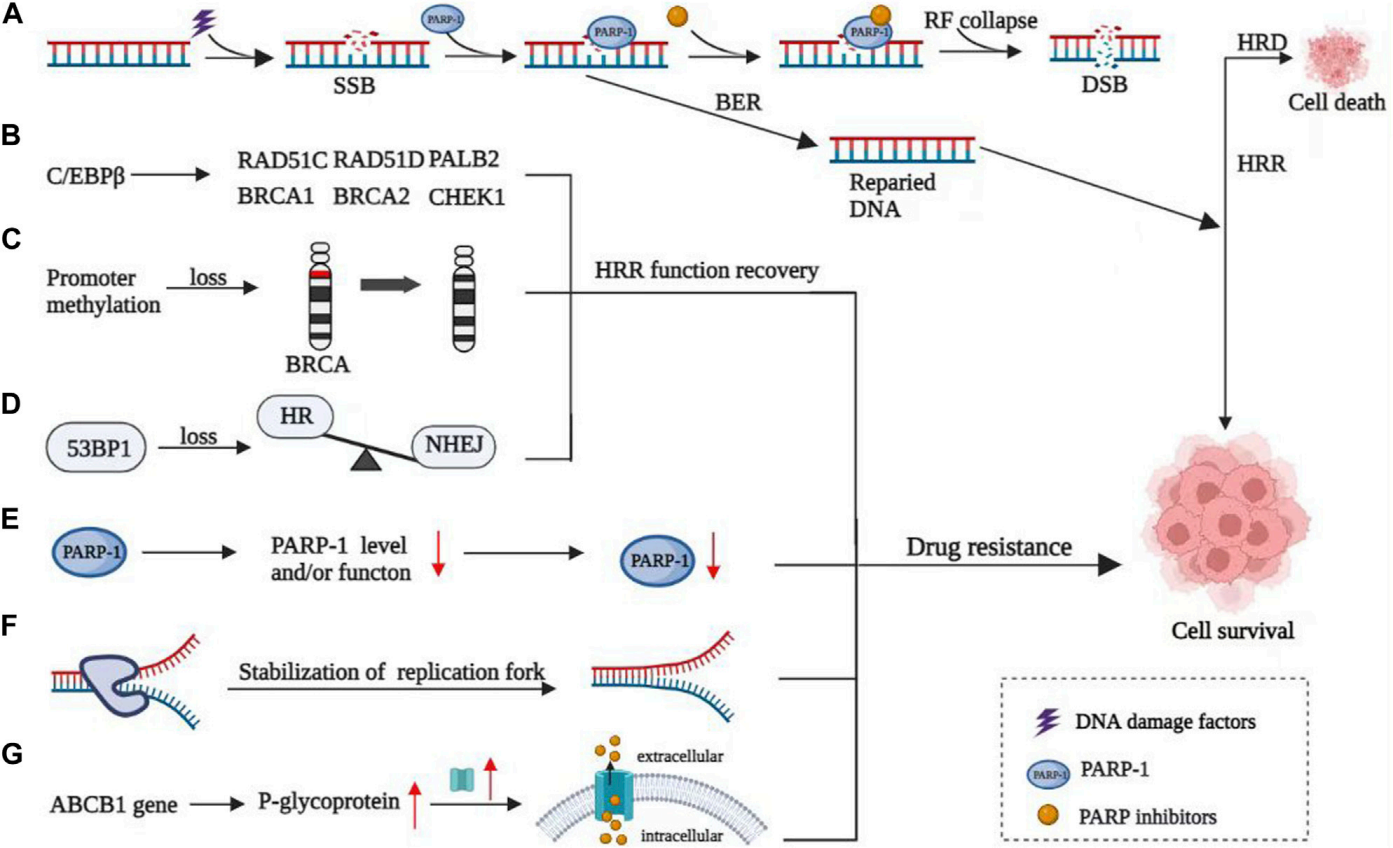
Mechanisms of resistance to PARP inhibitors. **(A)** Mechanism of PARP inhibitors. **(B)** C/EBPβ directly targeted and upregulated multiple HR genes, thereby inducing restoration of HR capacity and mediating acquired PARPi resistance. **(C)** Reverse methylation of *BRCA* genes leads to HRR function recovery. **(D)** 53BP1 contributed to the imbalance of HR and non-homologous terminal junction (NHEJ). **(E)** Loss of function of PARP1. **(F)** Stability of the replication fork and ultimately leading to PARPi resistance. **(G)** Overexpression of P-glycoprotein, encoded by the *ABCB1* gene, leading to the increase of intracellular drug expulsion.

DNA damage repair defects exist in all kinds of tumor cells. It is one of the mechanisms of tumor initiation and tumor therapy. The protein encoded by the *BRCA* gene is involved in the repair of DNA double-strand damage through the homologous recombination (HR) pathway. Breast cancer 1/2 gene (*BRCA1/2*) and others involved in homologous recombination repair (HRR) gene mutation or function can cause homologous recombination deficiency (HRD), causing malignant transformation in cells (Chiappa et al., 2021). Through the “synthetic lethality” mechanism, poly(ADP-ribose) polymerase inhibitor (PARPi) blocks the repair of DNA single-strand breaks in tumor cells with HRD, accumulating a large number of DNA double-strand breaks (DSBs), leading to the death of tumor cells, and thus showing significant anti-tumor activity in patients with HRR dysfunction (Kim et al., 2021). PARPi has emerged as a molecularly targeted therapeutic strategy for OC. Studies have shown that PARPis can significantly improve the progression-free survival (PFS) and overall survival (OS) of OC, especially in newly diagnosed and recurrent OC patients with BRCA mutations (Gong et al., 2020; Kim et al., 2021; Spiegel et al., 2021). Thus, PARPis have been widely used in BRCA-mutated (BRCAm) OC (Gong et al., 2020; Spiegel et al., 2021). Currently, four different PARPis have been approved by the US Food and Drug Administration (FDA) to treat OC, including olaparib, niraparib, rucaparib, and talazoparib (Xu and Li, 2021). PARPi has been shown to effectively increase PFS and OS in a broad population. While the intrinsic or acquired drug resistance of PARPi has gradually become an important clinical problem, this review summarizes the mechanisms of PARPi resistance (Figure 1), the current progress on combination strategies to overcome PARPi resistance, and the evaluation of PARPi resistance.

## 2 Mechanisms of resistance to PARPi

### 2.1 Reactivation of HR

Restoration of HRR function is an important mechanism for the PARPi resistance. It can be achieved through reversion mutations of HRR-related genes, reverse methylation of BRCA genes, and imbalance of HR and non-homologous terminal junction (NHEJ) caused by the deletion of p53 binding protein 1 (53BP1) and related effector molecules.

#### 2.1.1 Reversion mutations of HRR-related genes

The reversion mutations of HRR-related genes include BRCA1, BRCA2, RAD51C, RAD51D, and PALB2, which lead to the reactivation of the HRR function (Castroviejo-Bermejo et al., 2018; Nesic et al., 2021; Darabi et al., 2022). CCAAT/enhancer-binding protein β (C/EBPβ) is a transcription factor and also a key regulator of the HR pathway. C/EBPβ targeted and upregulated multiple HR genes, inducing restoration of HR capacity and mediating acquired PARPi resistance. PARPi responsiveness was inversely correlated with the expression of C/EBPβ in HR-proficient conditions, both *in vitro* and *in vivo*. High C/EBPβ expression enhanced PARPi resistance. PARPi treatment in turn induced C/EBPβ expression (Tan et al., 2021). Targeting C/EBPβ might induce HR deficiency and overcome PARPi resistance accordingly. Some mutations revert to the phenotype of the original wild-type gene, known as reversion mutations, which are the most important of the secondary mutations. Related mutations of *BRCA* genes mainly include pathogenic site restoration mutation, deletion or insertion of pathogenic site region leading to the reopening of gene open reading frame, exon mutation leading to the generation of splice variants, and primary mutation leading to drug resistance. Many cases have shown that BRCA-related reversion mutations can be observed after tumor progression by secondary test of circulating tumor cell DNA (ctDNA) in patients, who are with tumor progression during PARPi treatment, compared with before treatment initiation. Continuous collection of ctDNA can provide clues about the significance of individualized treatment for patients with tumor progression after PARPi treatment (Tan et al., 2021). For example, patients with reversion mutations in the *BRCA* gene in their secondary tests may be insensitive to PARPi. It is also supported by clinical evidence that reversion mutations in *BRCA* genes lead to PARPi resistance (Lin et al., 2019).

#### 2.1.2 Reverse methylation of *BRCA* genes

HRR function recovery can also be achieved by reversing *BRCA* gene promoter methylation. Loss of promoter methylation leads to increased expression of proteins related to DNA damage repair. It improves the DNA damage repair ability of drug-resistant cells and invalidates the effect of PARPi, leading to drug resistance (Ter Brugge et al., 2016; Weigelt et al., 2017).

#### 2.1.3 Imbalance of HR and non-homologous terminal junction (NHEJ)

53BP1 interacts with p53 to repair DNA damage and prevent the occurrence of tumors. 53BP1 plays an important role in the balance between HR and NHEJ. The study found that 53BP1 deletion restored HRR activity in cells with *BRCA1* gene mutation, possibly because 53BP1 deletion promoted the processing of terminal DNA to form single-stranded DNA and initiate HRR (Nacson et al., 2018). In the setting of 53BP1 KO, hypomorphic BRCA1 proteins are activated downstream of end resection, which promotes RAD51 loading and PARPi resistance. While in BRCA2-mutated cells, 53BP1 deletion does not cause PARPi resistance (Cruz et al., 2018).

#### 2.1.4 Loss of function of PARP1

The main targets of PARPi are polyadenosine diphosphate ribose polymerase (PARP) 1 and PARP2. The function change caused by mutations of these two targets will cause PARPi resistance. Studies have found that the mutation of the *PARP1* gene can affect the ability of PARP1 to bind DNA damage sites, resulting in reduced capture ability of PARP and reduced binding of PARPi, leading to drug resistance (Pettitt et al., 2018). In addition to the point mutation of the *PARP* gene, other factors such as the high expression of proto-oncogene c-Met mediate PARP1 phosphorylation, resulting in increased enzyme activity of PARP1 and reduced PARPi binding, leading to drug resistance of PARPi (Pettitt et al., 2018).

### 2.2 Increased drug efflux

Overexpression of the drug efflux protein is also considered to be one of the causes of PARPi resistance (Biegala et al., 2021). One study of recurrent high-grade ovarian serous cancer found that P-glycoprotein (P-gp) was overexpressed in about 56% of recurrent patients. P-gp is also called multi-drug resistance protein 1 (MRD1), encoded by the *ABCB1* gene. Its overexpression will lead to the increase of intracellular drug expulsion and the decrease of uptake. Thus, the intracellular drug concentration is reduced, and the result is PARPi resistant (Incorvaia et al., 2017).

### 2.3 Poly(ADP-ribose) glycohydrolase (PARG)

Poly(ADP-ribose) glycohydrolase (PARG) reverses the action of PARP enzymes by hydrolyzing PAR ribose bonds following DNA damage. Similarly, the positive role of PARG in DNA replication and repair leads to increased sensitivity of PARG-deficient cells to DNA-damaging agents. PARG-deficient cells presented reduced efficiency of double-strand break (DSB) and single-strand break (SSB) repair (Biegala et al., 2021). It suggests PARG could be a potential target in OC (Matanes et al., 2021).

### 2.4 Stabilization of the replication forks

In the special anti-tumor mechanism that replication forks protect PARPi, sufficient fatal DNA damage must be formed to start the apoptosis process. Thus, DNA damage, especially the reduction of fatal DNA damage, is one of the important reasons for PARPi drug resistance. In addition, the increased stability of replication forks is one of the important reasons for the reduction of DNA damage (Kharat et al., 2020). Most typically, the deletion of Pax2 reduces the recruitment of meiotic 11 (MRE11) to the stagnant replication fork. It avoids degradation of the replication fork, maintains the stability of the replication fork, and ultimately leads to PARPi resistance (He et al., 2018).

### 2.5 Miscellaneous mechanisms

The Wnt signaling pathway is a group of signal transduction pathways provoked by the binding of ligand proteins Wnt and membrane protein receptors. When this pathway is abnormally activated, β-catenin expression increases, promoting tumor growth, invasion, and metastasis. This is associated with the formation of platinum resistance and reduced olaparib and rucaparib sensitivity. Pyrvinium pamoate, an inhibition of Wnt signaling, can downregulate the expression of β-catenin (Hu et al., 2021). The use of pyrvinium pamoate can overcome PARPi-resistance. Yamamoto et al. (2019) found that the excessive activation of the Wnt/β-catenin signaling pathway is associated with PARP resistance in tumor cells (Yamamoto et al., 2019).

## 3 Strategies to reverse resistance

### 3.1 Replace other PARPis

Different PARPis have different cell membrane penetration, which affect the drug concentration in tissues. Compared with olaparib, the apparent permeability (PAPP) of niraparib is higher. Therefore, niraparib has higher cell membrane penetrability. Niraparib in OC tissue and intracranial drug concentration is higher (Sun et al., 2018). Correspondingly, the percentages of the CSF/plasma concentration (AUC 1–4h) in animal models administered with different doses of PARPi (pamiparib 10 mg/kg, niraparib 50 mg/kg, and olaparib 50 mg/kg) orally were 18%, 9%, and 2%, respectively. Thus, compared with other PARPi, pamiparib has higher membrane permeability and can better penetrate the blood–brain barrier (Xiong et al., 2020). Pharmacological differences may partly explain why PARPi may still be effective when replaced with another PARPi. The OReO/ENGOT-Ov38 study, conducted in 2017, is a randomized, double-blind, placebo-controlled multicenter phase IIIb clinical trial. It was the first to assess tumor progression on or after PARPi maintenance therapy. This study suggests that patients who respond to initial maintenance therapy can still benefit significantly from restarting the same PARPi maintenance therapy after a period of PARPi resistance discontinuation (Tew et al., 2020).

Upregulation of p-glycoprotein expression is one of the potential resistance mechanisms of PARPi. The selection of a kind of PARPi with a non-p-glycoprotein substrate can avoid the reduction of intracellular drug concentration caused by the drug pump, thus reducing the occurrence of drug resistance. Pamiparib is the only kind of PARPi with a non-p-glycoprotein substrate at present, which has certain anti-drug resistance (Xiong et al., 2020). This may be related to its unique drug structure and pharmacokinetics. At present, further translational medicine and clinical research data are needed to overcome PARPi resistance in tumor cells caused by drug efflux.

### 3.2 Anti-angiogenic agents

Studies have found that anti-angiogenic drugs can cause cell hypoxia and induce the downregulation of the HRR signaling pathway leading to HRD. It suggests that there may be some clinical benefit from the combination of antiangiogenic drugs and PARPi in patients with tumor progression after PARPi maintenance therapy (Lin et al., 2018; Bizzaro et al., 2021). A phase II clinical trial (NCT02354131) (Mirza et al., 2019) investigated the efficacy of compared niraparib and bevacizumab versus niraparib alone in platinum-sensitive recurrent OC. They found a significant improvement in PFS in the combination group compared with nilaparib alone (11.9 months vs. 5.5 months). Patients received oral niraparib 300 mg alone once daily or with intravenous bevacizumab 15 mg/kg once every 3 weeks until disease progression. EVOLVE study (Lheureux et al., 2020), published in 2020, is a multicenter, open, single-arm phase II trial of retreatment with cediranib combined with olaparib after PARPi resistance. A total of 34 patients with high-grade serous OC who relapsed or progressed after PARPi maintenance therapy were enrolled in the study. They were divided into a platinum-sensitive group, a platinum-resistant group, and a progression group after standard chemotherapy. Patients received olaparib tablets 300 mg twice daily with cediranib 20 mg once daily until progression or unacceptable toxicity. The study endpoint was a 16-week PFS rate, and 19% of the patients had reversion mutations in *BRCA1*, *BRCA2*, and *RAD51B* genes. In addition, 16% had *CCNE1* gene amplification, 15% had *ABCB1* gene upregulation, and 7% had *SLFN11* gene downregulation. The 16-week PFS rates of the three groups were 55%, 50%, and 39%, respectively. The patients with HRR gene reverse mutation and *ABCB1* gene upregulation had a poor prognosis. Subsequent treatments should be selected according to the HRR status. So, secondary genetic testing for PARPi-resistant patients is helpful for the selection of clinical treatment and the adjustment of the use of PARPi. In PARPi-resistant patients, the combination of antiangiogenic drugs and PARPi remains effective. The possible mechanism is that the antiangiogenic drug cediranib inhibits the expression of *BRCA1/2* and *RAD51* genes by transcriptional inhibition and inducing hypoxia (Pham et al., 2021).

### 3.3 Immune checkpoint inhibitors (ICIs)

In preclinical and early-stage clinical studies, PARPi has been found to enhance the response rate of immunotherapy by inhibiting DNA repair and producing DNA damage, promoting neoantigen release, increasing tumor mutation load, and enhancing programmed cell death ligand 1 (PD L1) expression (Maiorano et al., 2022). Whether the combination of PARPi and immune checkpoint inhibitor (ICI) can produce synergistic effects depends on two premises. One is the ability to increase DNA damage and produce cytoplasmic DNA. If PARPi resistance is caused by HRR function recovery, this premise is weakened. The other is the multiple immunoregulatory effects of PARPi, including T-lymphocyte differentiation, macrophage polarization, increased susceptibility to natural killer cell-mediated death, and upregulation of PD L1 (Chiappa et al., 2021). ICI can improve tumor sensitivity to PARPi. PARPi can induce tumor response to ICI by exacerbating DNA damage. Relevant clinical trials have been conducted and obtained good results. The phase I/II clinical trial (NCT02657889) (Konstantinopoulos et al., 2019) assessed the safety and efficacy of nilaparib in combination with pembrolizumab in patients with recurrent OC and determined the recommended phase II dose (RP2D) for clinical trials. RP2D was 200 mg of oral niraparib once daily and 200 mg of intravenous pembrolizumab on day 1 of each 21-day cycle. The MEDIOLA trial (NCT02734004) (Domchek et al., 2020) evaluated the safety and activity of olaparib in combination with dulvalumab in patients with BRCA2-mutated metastatic breast and OC. It showed that, among 32 patients with OC, the 28-week disease control rate (DCR) was 65.6% and the ORR was 71.9%, with seven patients achieving complete remission (CR). In the MEDIOLA trial, patients received 300 mg olaparib in tablet form orally twice daily for 4 weeks and thereafter a combination of olaparib 300 mg twice daily and durvalumab 1.5 g via intravenous infusion every 4 weeks until disease progression. In addition, PARPi combined with immune checkpoint inhibitors may still be effective in restoring the efficacy of HRR (Peyraud and Italiano, 2020).

### 3.4 Cell cycle checkpoint inhibitors

WEE1 is a regulatory molecule of the G2/M phase of the cell cycle checkpoint. When activated, WEE1 leads to G2/M cell cycle arrest and CDK1 phosphorylation, thus preventing HRR. Preclinical evidence suggests that this combination therapy has a synergistic effect (Haynes et al., 2018). In 2021, ASCO reported a phase II study evaluating Wee1 inhibitor (Adavosertib) monotherapy or a combination of PARPi in PARPi-resistant OC. The results showed that both the combination group and the monotherapy group were effective in PARPi-resistant OC (ORR 29%vs.23%) (Lheureux et al., 2021). As an activating protein of WEE1, CHK1 is another target that can be combined with PARPi. A phase I combination study of the CHK1 inhibitor prexasertib and the PARP inhibitor olaparib in HGSOC showed that four of 18 patients with BRCA1-mutant, PARPi-resistant HGSOC, achieved partial responses (Do et al., 2021). This study followed a 3 + 3 design with a 7-day lead-in of olaparib alone, followed by 28-day cycles with prexasertib administered on days 1 and 15 in combination with an attenuated dose of olaparib on days 1–5 and 15–19. Another phase I trial of CHK1 inhibitors in combination with PAPR inhibitors is also underway in OC with BRCA mutations that have previously received PAPR inhibitors (NCT03057145) (Smith et al., 2021). ATR is the upstream pathway of CHK1, and ATR is present in PARPi-resistant BRCA mutant cells. ASCO reported a study of PARPi combined with ATR inhibitor (ceralasertib) in PARPi-resistant relapsed OC, which showed clinical benefit (ORR 46%) (Shah et al., 2021).

## 4 Prediction of PARPI sensitivity

Cell-free DNA (cfDNA) is mainly released after cell necrosis, and most of them are derived from normal cells. In cancer patients, cfDNA is partially derived from tumor cells, and it also can be derived from other cells in the tumor microenvironment. CfDNA from dying tumor cells can metastasize to nearby cells and cells of distant organs and induce DNA damage and inflammatory responses through genomic integration (Hu et al., 2022). Weigelt et al. studied cfDNA from 24 prospectively accrued BRCA1- or BRCA2-germline mutant patients. They included 19 platinum-resistant/refractory OC and five platinum and/or PARP inhibitor pre-treated metastatic breast cancer patients. They found that diverse and often polyclonal putative BRCA1 or BRCA2 reversion mutations were identified in cfDNA from four OC patients (21%) and two breast cancer patients (Weigelt et al., 2017). BRCA reversion mutations detected can predict drug resistance to rucaparib in HGSOC patients. This suggests cfDNA might be an effective way for evaluating and monitoring resistance of olaparib (Lin et al., 2019; Hu et al., 2022).

## 5 Future directions

PARPi maintenance therapy is a crucial part of the whole treatment management of OC. The emergence of PARPi has brought great innovation in targeted therapy of OC. Like chemotherapeutic drugs, drug resistance of PARPi is an inevitable clinical challenge. The mechanisms of PARPi resistance are complex and diverse. Secondary testing of cfDNA and related genes can help screen people who are likely to benefit again and formulate re-maintenance treatment. Overcoming PARPi resistance depends on the combination with other drugs (Table 1). In addition, the appropriate dose and administration of each drug should be determined to minimize adverse events while ensuring maximum benefits and outcomes. However, most of the combination treatment schemes are only in the early stage of clinical trials. Further exploration and more clinical practice data are needed to support the dose, toxicity, side effects, efficacy, and population screening.

**TABLE 1:** Overview of clinical trials assessing treatments for ovarian cancer patients resistant to PARP inhibitors.

Category	Study name or identifier	Phase	PARPi	Combination	Population	Results	Study status
Replace other PARPis	OReO/ENGOT-Ov38 NCT03106987	IIIb	Olaparib	-	Patients with epithelial ovarian cancer previously treated with a PARPi and responding to repeat platinum chemotherapy	PFS 5.3 months vs. 2.8 months (olaparib group vs. placebo group)	Completed
Antiangiogenic agents	NCT02354131	II	Niraparib	Bevacizumab	Platinum-sensitive recurrent ovarian cancer	PFS 11.9 months vs. 5.5 months (combination group vs. nilaparib alone)	Completed
	EVOLVE	II	Olaparib	Cediranib	Patients with high-grade serous ovarian cancer who relapsed or progressed after PARPi maintenance therapy	16-week PFS rate 55%, 50%, and 39%, respectively (platinum-sensitive group, platinum-resistant group, and progression group after standard chemotherapy)	Completed
Immune checkpoint inhibitors (ICIs)	NCT02657889	I/II	Nilaparib	Pembrolizumab	Patients with recurrent ovarian cancer	ORR was 18% (90% CI, 11%–29%), with a disease control rate of 65% (90% CI, 54%–75%), including three (5%) with confirmed complete responses, eight (13%) with confirmed partial responses, 28 (47%) with stable disease, and 20 (33%) with progressive disease	Completed
	MEDIOLA NCT02734004	I/II	Olaparib	Dulvalumab	Patients with BRCA2-mutated metastatic breast and ovarian cancer	The 28-week disease control rate (DCR) was 65.6%, and the ORR was 71.9%, with 7 patients achieving complete remission (CR)	Recruiting
Cell cycle checkpoint inhibitors	NCT03057145	I	Olaparib	Prexasertib	Advanced solid tumors	Four of 18 BRCA1-mutant patients who were P ARPi-resistant achieved a partial response	Completed

## 6 Conclusion

The selection of potential combination drugs is the key to overcoming PARPi resistance. The most effective combination regimen for PARPi-resistant patient populations remains unclear. Predicting and assessing adaptive responses to genomic or epigenetic changes may help rationally select combination therapies and avoid acquired resistance. The ultimate clinical goals are to improve the prognosis of OC, optimize the combination therapy based on specific tumor molecular mechanisms, and achieve the synergistic effect of drugs.
